# A novel *IRF6* gene mutation impacting the regulation of *TGFβ2-AS1* in the TGFβ pathway: A mechanism in the development of Van der Woude syndrome

**DOI:** 10.3389/fgene.2024.1397410

**Published:** 2024-06-05

**Authors:** Zhiyang Zhao, Renjie Cui, Haoshu Chi, Teng Wan, Duan Ma, Jin Zhang, Ming Cai

**Affiliations:** ^1^ Department of Oral and Craniomaxillofacial Surgery, Shanghai Ninth People’s Hospital, Shanghai Jiao Tong University School of Medicine, College of Stomatology, Shanghai Jiao Tong University, National Center for Stomatology, National Clinical Research Center for Oral Diseases, Shanghai Key Laboratory of Stomatology, Shanghai Research Institute of Stomatology, Shanghai, China; ^2^ Department of Molecular Diagnostics & Endocrinology, The Core Laboratory in Medical Center of Clinical Research, State Key Laboratory of Medical Genomics, Shanghai Ninth People’s Hospital Affiliated to Shanghai Jiao Tong University School of Medicine, Shanghai, China; ^3^ Shanghai Xuhui District Dental Disease Center, Shanghai, China; ^4^ Key Laboratory of Metabolism and Molecular Medicine, Ministry of Education, Department of Biochemistry and Molecular Biology, Collaborative Innovation Center of Genetics and Development, Institutes of Biomedical Sciences, School of Basic Medical Sciences, Shanghai Medical College, Fudan University, Shanghai, China

**Keywords:** Van der Woude syndrome, IRF6, TGFβ signaling pathway, epigenetics, orofacial clefts

## Abstract

Several mutations in the *IRF6* gene have been identified as a causative link to VWS. In this investigation, whole-exome sequencing (WES) and Sanger sequencing of a three-generation pedigree with an autosomal-dominant inheritance pattern affected by VWS identified a unique stop-gain mutation—c.748C>T:p.R250X—in the *IRF6* gene that co-segregated exclusively with the disease phenotype. Immunofluorescence analysis revealed that the *IRF6*-p.R250X mutation predominantly shifted its localization from the nucleus to the cytoplasm. WES and protein interaction analyses were conducted to understand this mutation’s role in the pathogenesis of VWS. Using LC-MS/MS, we found that this mutation led to a reduction in the binding of IRF6 to histone modification-associated proteins (NAA10, SNRPN, NAP1L1). Furthermore, RNA-seq results show that the mutation resulted in a downregulation of TGFβ2-AS1 expression. The findings highlight the mutation’s influence on *TGFβ2-AS1* and its subsequent effects on the phosphorylation of SMAD2/3, which are critical in maxillofacial development, particularly the palate. These insights contribute to a deeper understanding of VWS’s molecular underpinnings and might inform future therapeutic strategies.

## 1 Introduction

Orofacial clefts (OFC) are the predominant craniofacial deformities observed in humans and are categorized into syndromic and non-syndromic clefts depending on the genetic phenotype. The most common genetic form of OFC is Van der Woude syndrome (VWS), which accounts for approximately 2% of all cases, with a prevalence estimated at 1 in 35,000 individuals (Leslie et al., 2013). VWS is characterized as an autosomal dominant condition with remarkable penetrance (96.7%) that is manifested in a spectrum of clinical presentations encompassing lower lip pits/fistulae, cleft lip/palate, and submucous cleft palate (Van Der Woude, 1954; Wang et al., 2019).

To date, mutations to three pivotal genes have been associated with VWS etiology: interferon regulatory factor 6 (*IRF6*), Grainyhead-like transcription factor (GRHL3), and a scarcely observed pathogenic missense mutation in NME/NM23 nucleoside diphosphate kinase 1 (NME1) (Malik et al., 2014; Peyrard-Janvid et al., 2014; Parada-Sanchez et al., 2017). *IRF6* is distinguished as a member of the IRF transcription factor family that has been singularly implicated in craniomaxillofacial morphogenesis (Starink et al., 2017). The IRF family is unified by a conserved helix-turn-helix DNA-binding domain (DBD) that is complemented by a variably conserved protein-binding region denoted as the Ski-interacting protein-like domain (SMIR). A repertoire of over 200 mutations in *IRF6*, spanning missense, non-sense, frameshift, microdeletions, and splice-site mutations, has been documented in relation to VWS (Zhao et al., 2018; Wang et al., 2019; Yu et al., 2020). Collectively, these mutations account for 72% of VWS diagnoses. Notably, their distribution is not random, with a significant proportion being localized to either exons 3 and 4, which contain the gene encoding the DBD, or exons 7 and 9, which contain the gene encoding the protein-binding domain (de Lima et al., 2009; Charzewska et al., 2015; Busche et al., 2016; Bennun et al., 2018). IRF6 is regulated by its upstream TGFβ3, and it not only regulates downstream TGFβ2 but also modulates the phosphorylation of SMAD2/3. Ultimately, by modulating the TGFβ signaling pathway, IRF6 participates in the development of the maxillofacial region, particularly the palate (Ke et al., 2015; Nakajima et al., 2018; Ke et al., 2019; AlMegbel and Shuler, 2020).

*TGFβ2-AS1* is a multifunctional lncRNA that plays a significant role in the TGFβ signaling pathway and is capable of both upregulating and downregulating various genes and influencing the pathway at multiple levels, from epigenetic regulation to modulation of phosphorylation events (Papoutsoglou et al., 2019). Its complex interactions and regulatory mechanisms make it a significant factor in cellular signaling and a potential target for therapeutic intervention.

In this study, we assembled a family tree of Chinese Han individuals with VWS and conducted whole-exome sequencing (WES) to identify potential genetic underpinnings that could be implicated in the development of VWS in this pedigree. During this analysis, we identified a unique stop-gain mutation—c.748C>T:p.R250X—in the *IRF6* gene, which had not been previously reported. Therefore, we assessed the impact of the mutation on the cellular localization of IRF6 and the binding of IRF6 to other proteins, including several histones. Moreover, we also evaluated the impact of the mutation on the expression of *TGFβ2-AS1* and phosphorylation of SMAD2/3 to determine how the mutated *IRF6* gene would affect the activity of the TGFβ signaling pathway.

## 2 Materials and methods

### 2.1 Clinical samples

The study received approval from the local ethics and research committee of the Shanghai Ninth People’s Hospital (SH9H-2021-T357-2). In 2021, the Ninth People’s Hospital, affiliated with the Shanghai Jiaotong University School of Medicine, initiated the recruitment and subsequent enrollment of a three-generation, seven-member family presenting with VWS as well as unrelated control subjects. Before the commencement of any research-related activities, written informed consent was secured from all participants. For individuals under 18 years of age, informed consent was received from their respective guardians in strict adherence to the tenets of the Declaration of Helsinki. Physical exams were conducted by two surgeons to examine any possible organ malformations. The proband’s mother underwent a detailed review of her prenatal exposure history, encompassing factors such as smoking habits, alcohol consumption, medication and supplement intake, prior illnesses, and potential radiation exposure.

### 2.2 Whole-exome sequencing (WES)

To pinpoint potential pathogenic gene mutations within the pedigree, we conducted WES of the genomic DNA isolated from the family members. Venous blood (2 mL per individual) was drawn from each participant and anticoagulated with EDTA. Subsequent genomic DNA isolation was facilitated using a Qiagen DNA extraction kit according to the manufacturer’s recommended protocol. Amplification of the genes of interest was performed by polymerase chain reaction (PCR) using primers encompassing all exonic and adjacent intronic regions (primer sequences available upon request). WES was carried out on a BGISEQ-500 platform (BGI, China) to ascertain the underlying mutation. The data from each sample underwent sequence alignment to the human reference genome (GRCh37/hg19) utilizing the Burrows-Wheeler Aligner tool (Oxford, UK). Mutation discernment was facilitated via the Genome Analysis Toolkit (GATK; accessible at https://www.broadinstitute.org/gatk/guide/best-practices). Metrics encompassing sequencing depth and coverage for each specimen were deduced from the alignment outputs. Annotations and pathogenicity predictions were subsequently orchestrated through SnpEff (detailed at http://snpeff.sourceforge.net/SnpEff_manual.html).

### 2.3 Sanger sequencing

Primer3 (https://bioinfo.ut.ee/primer3/) was used to design primers for the identified pathogenic mutations. These primers were then added to the samples from the VWS patients and healthy individuals, and the target genes were amplified using PCR. The samples then underwent Sanger sequencing to compare the obtained results with the reference sequence (NM_006147.4).

### 2.4 Antibodies

The antibodies used in our study were procured from two main suppliers: Abmart and Cell Signaling Technology. From Abmart, we obtained the mouse monoclonal antibody targeting DYKDDDDK-Tag (3B9) (M20008S) for Western blot, an anti-EGFP antibody (PS09757S), Alexa Fluor 488-labelled goat anti-mouse IgG (M21011L), and Alexa Fluor 594-labelled goat anti-rabbit IgG (M21014L) for immunofluorescence. Cell Signaling Technology provided us with rabbit antibodies against SMAD3 (9523S), phospho-SMAD3 (9520S), phospho-SMAD2 (18338S), and GAPDH mouse antibodies (2118S) as well as HRP-linked anti-rabbit IgG (7074P2) and anti-mouse IgG (7076S) secondary antibodies.

### 2.5 Plasmids

The full-length *IRF6* sequence (NCBI Refseq: NM_006147.4) was amplified using standard PCR techniques and subsequently inserted into a pCDH-SBP-HIS_8_ vector. The mutations of *IRF6* were crafted through PCR and then integrated into the pCDH-SBP-HIS_8_ vector. Likewise, the full-length *TGFβ2-AS1* gene (NCBI Refseq: NG_027721.3) was synthesized using basic PCR and then subcloned into the pGreen-GFP-puro vector.

### 2.6 Cell culture and lentiviral transduction

Human oral keratinocytes (HOKs) isolated from human mucosa were purchased from ScienCell (Carlsbad, CA, United States) and maintained in Oral Keratinocyte Medium (ScienCell). The HOKs were inoculated into 10-cm Petri dishes containing Dulbecco Modified Eagle Medium (DMEM; Gibco, Carlsbad, CA, EUA) containing 4 mM l-glutamine, 1.5 g/L sodium bicarbonate and 4.5 g/L glucose and cultured at 37°C in a humidified atmosphere of 5% (v/v) CO2. The culture medium was supplemented with 10% fetal bovine serum (FBS), 100 μg/mL streptomycin, 100 μg/mL penicillin and 0.1% gentamicin. After culturing, the cells were washed twice with phosphate-buffered saline (PBS) to remove any impurities or residual media. The cells were then treated with 2 mL of 0.05% T/E solution and incubated for about 5 minutes until each cell morphed into a distinct spherical shape. This transformation is followed by centrifugation at 1,000 rpm for 3 minutes. The cells were then rejuvenated with fresh culture mediumand then transferred to a new dish and cultured at a density of 5,000 cells/cm^2^, ensuring optimal space for growth and nutrient absorption.

One of the intriguing experiments involved the lentivirus-induced overexpression of *IRF6* in HEK-293T cells. In this process, 4 ng of either wild-type *IRF6* or mutated *IRF6* pCDH-SBP-HIS_8_ plasmid, combined with 1 ng each of pCMVdelta8.2 packaging vector and VSVP enveloped vector, were co-transfected into HEK-293T cells housed in 60 mm cell-culture dishes from NEST. Following a 48 and 72-h incubation period, the virus-laden supernatant was carefully harvested. The supernatant was collected by filtration through a 0.45 mm PES syringe filter (Thermo Fisher, United States) to ensure purity. The cells were then exposed to this lentivirus in the presence of 8 mg/mL polybrene (Sigma-Aldrich, St. Louis, MO, United States) to initiate the infection process. In the final phase of lentiviral infection, the HOK cells demonstrating positive signs of infection were cultured in 5 mg/mL puromycin (Solarbio) for 5 days This ensured that only the cells proficiently expressing the desired traits were selected.

### 2.7 Immunofluorescence (IF)

After washing thrice with PBS, the HEK-293T cells overexpressing IRF6 were fixed with 4% paraformaldehyde for 10 min at room temperature, and the cell membranes were permeabilized with 0.2% Triton X-100 for 1 hour. Following incubation overnight at 4°C with the appropriate primary antibodies, the permeabilized cells were incubated with a goat anti-mouse FITC-conjugated secondary antibody for 1 hour at room temperature, after which the cells were stained with DAPI to visualize the cell nuclei. Aliquots of the cell mixtures were loaded onto microscope slides, which were supplemented with fluorescence decay-resistant medium (Beyotime) and mounted under a confocal laser-scanning microscope (Leica) for visualization.

### 2.8 RNA sequencing

Total RNA was extracted from a population of 1 × 10^6^ HOK cells using the TRIzol reagent (Invitrogen). The total RNA extracted from lentivirus-infected HOK cells constructed in the above steps was reverse transcribed into cDNA, and the cDNA was fragmented to construct the library. The QC-qualified libraries were sequenced (Illumina PE150), and clean data were filtered out after obtaining the raw data. Subsequently, the sequencing reads were aligned to the mouse reference genome (GRCm38/mm10) by HISAT2 software. The mRNA expression abundance was analysed using StringTie software, and differential expression analysis was performed on the transcript data of each group using DESeq2 R (version 1.10.1) software package. Gene set enrichment analysis (GSEA) and gene ontology (GO) enrichment analyses were performed on the screened differentially expressed genes to obtain the biological processes and signalling pathways that might be regulated by IRF6.

Biological variability was assessed by conducting principal component analysis (PCA) on the pre- and post-normalization data using the R package (version 3.6.1). Box plots using the ggplot2 package in R (version 3.6.1) indicated relatively uniform standardized data distribution. After standardization of the raw data, mRNAs of at least 5 out of 15 samples with current or edge markers were selected for further data analysis. *p*-values for assessing the differences in transcriptional levels were calculated using an unpaired t-test and then adjusted with the Benjamini–Hochberg FDR method. Differentially abundant mRNAs were then further filtered using an FC cutoff of >1.5 and an FDR cutoff of <0.05 to identify the DEGs. The volcano plot and heatmap of the DEGs were constructed using GraphPad Prism (version 8.0.1) and R/pheatmap package (version 1.0.12).

### 2.9 Mass spectrometry

HOK cells were stably transfected with pCDH-SBP-HIS_8_-*IRF6*-c.748C>T. The cells were lysed in NP-40 protein lysis buffer for 30 min at 4°C, and Western blotting was performed to quantify the expression levels of mutant IRF6. The SBP-His_8_-tagged mutant *IRF6* protein was precipitated by a two-step affinity purification over streptavidin-agarose resin and nickel resin columns. The precipitate containing the complex copurified with SBP-HIS_8_-*IRF6*-c.748C>T:p.R250X was digested with sequencing-grade trypsin (Promega). The resulting peptides were analyzed by LC-MS/MS in ESI mode using an Eksigent 2D nanoLC coupled in-line with an LTQ-Orbitrap mass spectrometer. More detailed steps were conducted as described previously (Beaty et al., 2010).

### 2.10 Western blot and co-immunoprecipitation (co-IP) assay

HOK cells were collected, washed twice with precooled PBS, and lysed in NP-40 protein lysis buffer at 4°C for 30 min. The protein concentration was measured using a bicinchoninic acid (BCA) assay kit (Thermo Fisher Scientific). Immunoblotting was conducted as previously described (Cui et al., 2022). For immunoprecipitation, total cell lysates were incubated with specific antibodies for 2 hours followed by rotation with flag beads overnight at 4°C. The resulting beads were washed three times with NP-40 lysis buffer and mixed with 1x SDS loading buffer before being boiled for 10 min. Finally, the supernatant was analyzed by immunoblotting.

### 2.11 Quantitative polymerase chain reaction (qPCR)

Total RNA was extracted from the HOK cells utilizing the TRIzol reagent (Thermo Fisher, 98597101) and reverse-transcribed into complementary DNA (cDNA) using the PrimeScript RT reagent kit with gDNA Eraser (YEASEN, H2107011). The quantitative PCR (qPCR) was performed on an ABI 7900 real-time PCR (Applied Biosystems) using the SYBR Premix Ex TaqTM II (Perfect Real Time, Takara, A8402-1); GAPDH served as an endogenous control to normalize gene expression levels. The raw data were analyzed. The 2^–ΔΔCT^ method was employed for accurate quantification of gene expression levels. Table 1 shows the sequences of the qPCR primers (Shanghai Sangon Biotechnology, Shanghai, China).

**TABLE 1:** Primer sequences.

Primer	Sequence (5’to3′)
TGFB2-AS1 F	GAG​TGT​GGA​AAT​GAG​GAC​CG
TGFB2-AS1 R	GAG​ATC​TTG​GGT​TTG​GGA​GT
CDH2 F	CAG​AGT​TTA​CTG​CCA​TGA​CG
CDH2 R	ATC​TCC​GCC​ACT​GAT​TCT​GT
TGFB2 F	GAA​TGG​CTC​TCC​TTC​GAT​GT
TGFB2 R	TGC​AGC​AGG​GAC​AGT​GTA​AG
CTGF F	GGT​TAC​CAA​TGA​CAA​CGC​CT
CTGF R	TTT​TGG​GAG​TAC​GGA​TGC​AC
GAPDH F	CAA​ATT​CCA​TGG​CAC​CGT​CA
GAPDH R	ATC​GCC​CCA​CTT​GAT​TTT​GG

### 2.12 Statistical analysis

All data reported herein represent as the mean ± standard error of the mean (SEM). Statistical significance was assessed using Student's t-test. Significance levels are denoted as follows: *p < 0.05, **p < 0.01, and ***p < 0.001, with the latter indicating the most statistically significant difference.

## 3 Results

### 3.1 Clinical information

**FIGURE 1:**
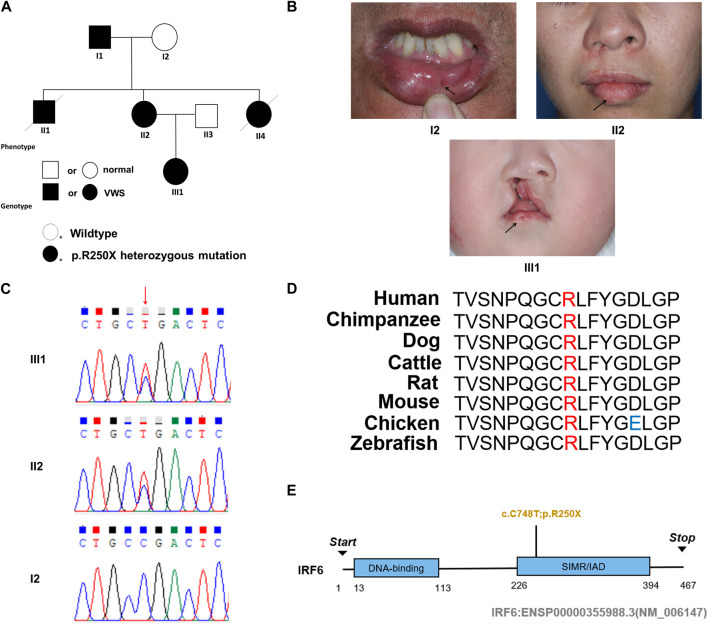
Pedigree information, phenotypes, and Sanger sequencing of the causative mutation in the VWS pedigree. **(A)** The solid black circle and square represent the patients with VWS. **(B)** The proband (III-1) is a girl with left complete cleft lip and palate. Her mother (II-2) had lower lip pits and repaired right cleft lip. Another patient (I-1) had left lower lip pits. The black arrow indicates the proband. **(C)** Sanger sequencing of the causative mutation. The mutations in II-1 and III-1 were heterozygous; I2 was the wild type. Red arrow indicates the position of causative mutation. **(D)** Protein sequence alignment of IRF6 orthologs was performed by Multiple Sequence Alignment (MUSCLE). The p. R250X mutation and the associated new residue sequence are shown above and indicated in red. **(E)** A schematic diagram of functional domains in the IRF6. The mutation was located in the second domain (IRF), p. R250X.

This study entails a detailed examination of a consanguineous family pedigree of three generations. The proband III:1 had a clinical diagnosis of VWS (Figure 1A). This diagnosis was primarily attributed to distinct physical anomalies: a complete cleft lip and a lower lip pit, both prominently exhibited on the right side of the face (Figure 1B). The analysis of the family pedigree revealed intricate patterns of inheritance and phenotypic manifestations spanning multiple generations. In the second generation, the family comprised three members (Figure 1A). In the second generation of the family line, II:1, II:2, and II:4 all had the disease, and all presented with a lower lip pit. However, both II:1 and II:4 died in childhood from other diseases, and the phenotype of the patients could not be further characterized. This continued into the third generation, as the prototype III:1 also presented with a right cleft lip. (Figure 1B). A scrutiny of the family’s generational health portrait unveiled a discernible autosomal-dominant pattern of inheritance. This pattern underscored the familial passage of the condition, laying bare a roadmap of genetic transmission and phenotypic expression that holds the promise of unlocking deeper insights into the enigmatic nature of this condition.

### 3.2 A novel mutation of *IRF6* gene leads to VWS

In this comprehensive study, WES was performed on three patients afflicted with a specific genetic disorder and one unaffected individual (Table 2). A total of 90 mutated genes were uncovered through this process. We employed Sanger sequencing to authenticate the disease-associated nonsense mutation, which included a hitherto unreported mutation of *IRF6* (c.748C>T:p.R250X) located in exon 7 (Figure 1C). This novel discovery revealed the stop-gain mutation situated within the SIMR/IAD-domain (Figure 1E). Notably, this mutation was not present in any of the unaffected family members and had not been previously cataloged in the dbSNP Database. A meticulous review of existing literature disclosed a significant association between diverse mutations within the *IRF6* gene and the onset of both cleft lip/palate and VWS. Our in-depth analysis of protein structure and functional prediction underscored the profound impact of this specific mutation. It instigates the transformation of the arginine (Arg) codon at position 250 within the *IRF6* gene into a stop codon, engendering a significant alteration in the resultant protein structure. In a comparative analysis spanning various species including *Homo sapiens*, *Mus musculus*, *Rattus norvegicus*, *Cavia porcellus*, *Pan troglodytes*, *Equus caballus*, *Papio anubis*, and *Oryctolagus cuniculus*, the Arg250 residue was found to be evolutionarily conserved (Figure 1D). The conservation of this residue across multiple species underscores its fundamental role, and mutations at this site could potentially have far-reaching implications for protein functionality and, by extension, the development and progression of associated genetic disorders. These findings lay a robust foundation for further research aimed at elucidating the intricate relationships between *IRF6* mutations and their clinical manifestations, paving the way for enhanced diagnostic precision and the development of targeted therapeutic interventions.

**TABLE 2:** Variant and clinical summary of pedigree.

Individual	Gender	Age (years)	Phenotype	Diagnosis	Genotype
I:1	M	56	Lip pit	VWS	p.R250X/wt
I:2	F	52	Normal	Normal	wt/wt
II:1	M	5	Lip pit	VWS	p.R250X/wt
II:2	F	24	Lip pit	VWS	p.R250X/wt
II:3	M	26	Normal	Normal	wt/wt
II:4	F	4	Lip pit	VWS	p.R250X/wt
III:1	F	1	Lip pit, cleft lip and cleft palate	VWS	p.R250X/wt

VWS: van der woude syndrome; wt: wild type.

### 3.3 The *IRF6*-c.748C>T:p.R250X mutation altered the localization of the wild-type full-length IRF6 protein

**FIGURE 2:**
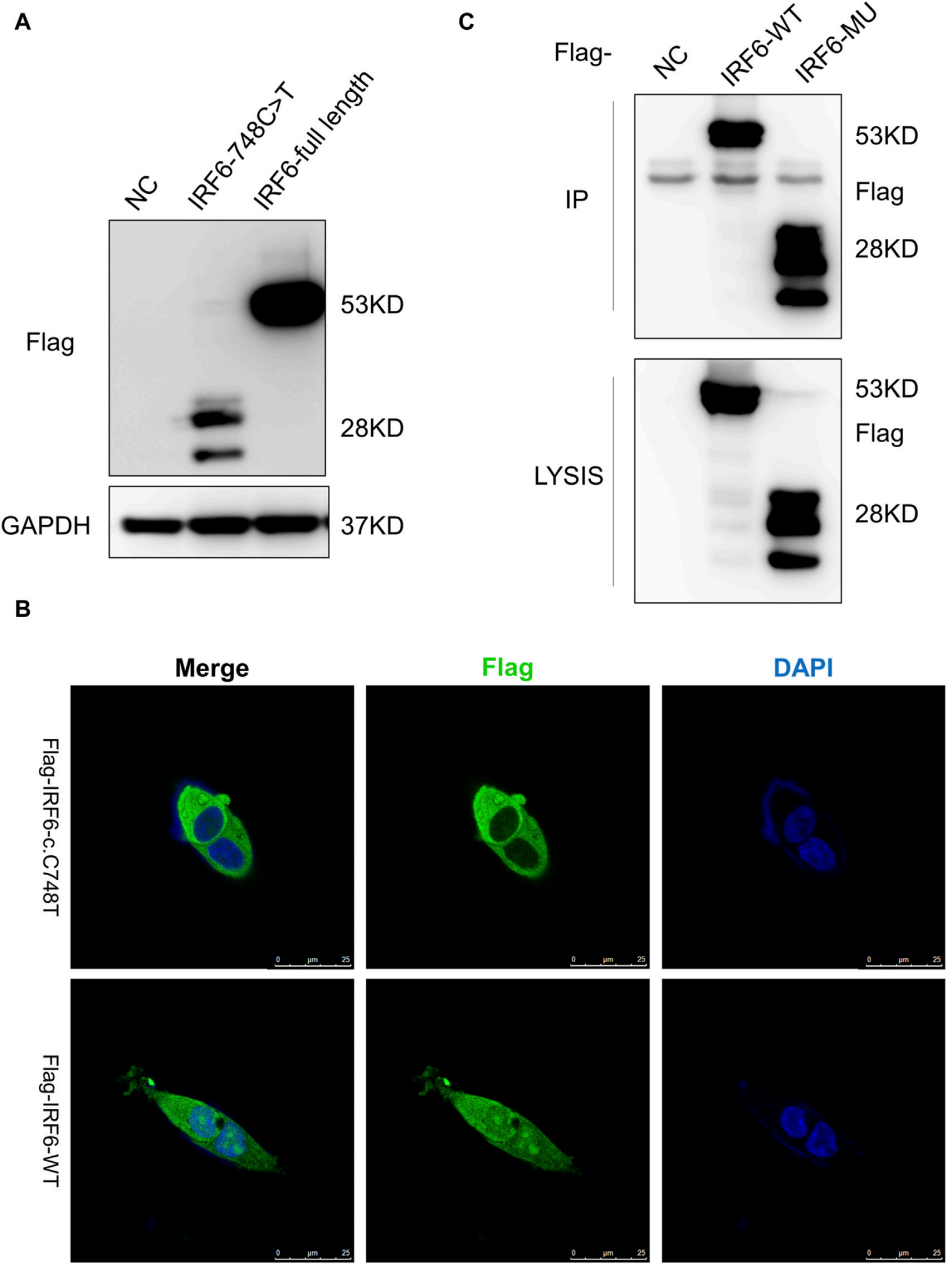
*IRF6*-c.748C>T:p.R250X mutation altered the protein localization and protein-binding partners. **(A)** HOK cells stably expressing Flag-tagged *IRF6*-c.748C>T:p.R250X or wild-type *IRF6* were developed by lentiviral infection and puromycin selection. **(B)** The *IRF6* localization was enabled by anti-flag immunofluorescence staining of the cells. The cells were stained with DAPI to visualize the nuclei. The truncated *IRF6*-c.748C>T:p.R250X was mainly localized to the cytoplasm, while the wild-type IRF6 was localized in the cytoplasm and the nucleus. Scale bar = 5 μm. **(C)** Flag-tagged *IRF6*-c.748C>T:p.R250X was immunoprecipitated from the cell lysate of HOK cells stably expressing Flag-tagged *IRF6*-c.748C>T:p.R250X, and the coimmunoprecipitation product was analyzed by anti-Flag immunoblotting.

HOK cells stably expressing Flag-tagged IRF6-c.748C>T:p.R250X or wild-type IRF6 were prepared by lentiviral infection and puromycin selection (Figure 2A). Immunofluorescence investigations revealed distinct localization patterns for the wild-type and mutated (c.748C>T:p.R250X) IRF6 in the HOK cells. Wild-type IRF6 was expressed in both the cytoplasm and nucleus, while the mutated IRF6 was predominantly localized to the cytoplasm (Figure 2B), indicating a potential impact on its nuclear import and functionality therein. We advanced our exploration to elucidate the disparity in protein interactions between the wild-type and mutated IRF6 using SBP-HIS tandem pull-down assays. These assays were also conducted with HOK cells stably expressing either SBP-HIS-tagged IRF6- c.748C>T:p.R250X or wild-type IRF6. Both cell types expressed the SBP-HIS-tagged protein, albeit the molecular weight of IRF6-c.748C>T:p.R250X was found to be lesser, indicating that mutation led to a truncating of the protein (Figure 2C).

Subsequent mass spectrometry analysis illuminated stark differences in protein-binding affinities. Of the 374 proteins that exhibited differential binding capacities to the mutant IRF6 in the mutant IRF6-expressing HOK cells compared to the wild-type IRF6, a refined list of 116 proteins was established post-exclusion of non-specific binding entities and proteins identified with less than two unique peptides. Detailed examination revealed that 52 proteins had diminished binding affinities to the mutant IRF6, whereas 27 exhibited enhanced binding in the context of the mutated IRF6. In the subset of proteins with reduced binding affinity, *N*-α-acetyltransferase 10 (NAA10), small ribonucleic acid polypeptide N (SNRPN), and ribosome assembly protein 1 (NAP1L1) were notably identified (Table 3). Additionally, further analysis revealed four phenotypically pertinent proteins—methylenetetrahydrofolate dehydrogenase, cyclase and formyltetrahydrofolate synthase 1 (MTHFD1), lysine-specific histone demethylase 1A (KDM1A), histone deacetylase 1 (HDAC1), and H2A.Z mutation histone 1 (H2AFZ)—whose binding capacity to mutant IRF6 was significantly lower compared to wild-type IRF6 (Table 4). These findings underscored the intricate molecular landscapes dictated by the *IRF6* mutations and their consequent impact on protein-protein interactions.

**TABLE 3:** Proteins with reduced binding capacity were obtained by pristine analysis.

↓	Protein, GN	IRF6-WT peptides	IRF6-MU peptides	Annotation
	NAA10	4	0	Histone acetylation
	SNRPN	2	0	Histone modification
	NAP1L1	2	0	Histone methylation modification

**TABLE 4:** Proteins with increased binding capacity were obtained by pristine analysis.

↑	Protein, GN	IRF6-WT peptides	IRF6-MU peptides	Annotation
	MTHFD1	1	2	Histone modification
	KDM1A	0	1	Histone demethylation
	HDAC1	0	1	Histone deacetylation
	H2AFZ	0	2	Heterochromatin formation

### 3.4 IRF6 affects the TGFβ signaling pathway by regulating the expression of TGFβ2-AS1

**FIGURE 3:**
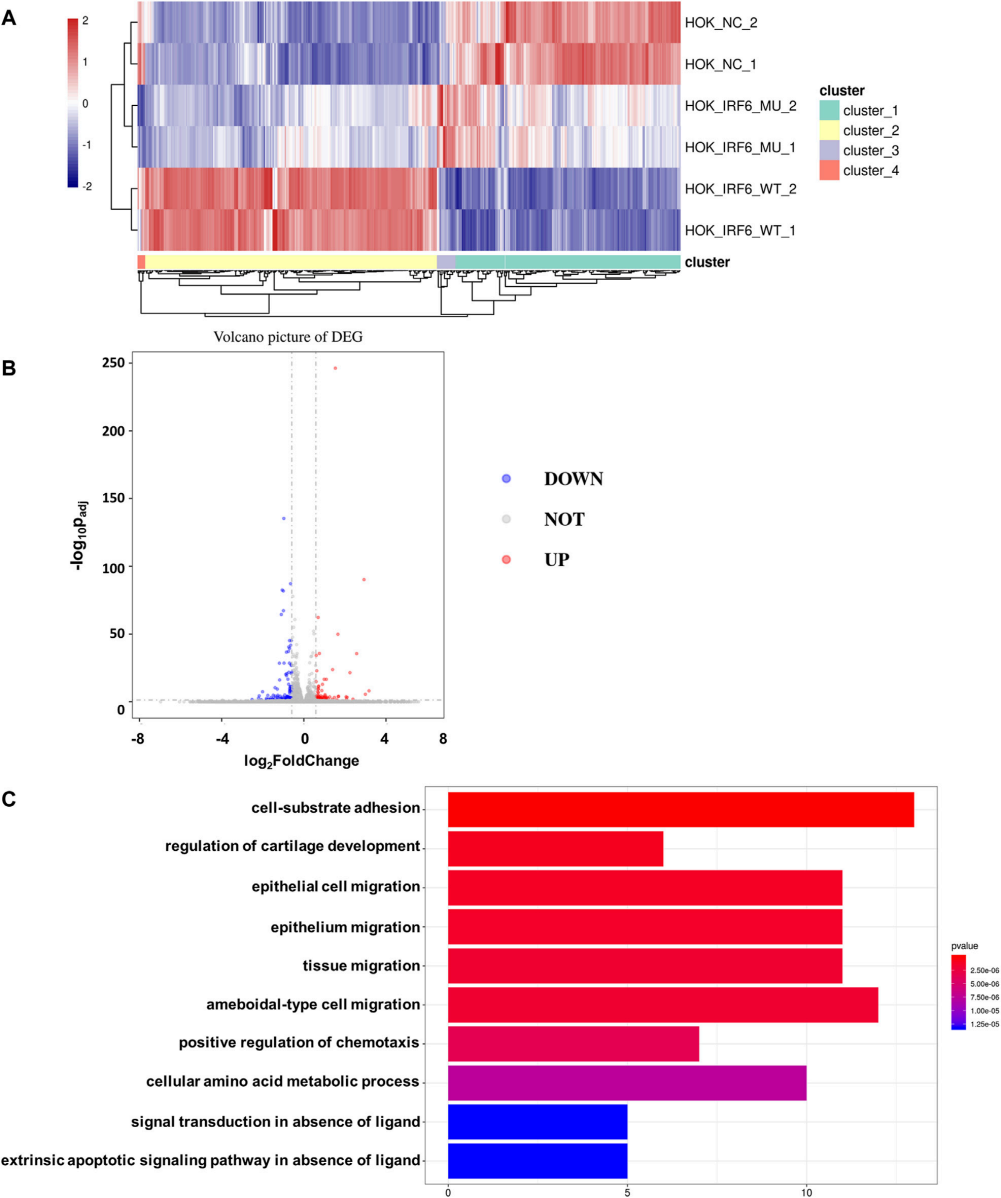
*IRF6*-c.748C>T:p.R250X mutation affected transcriptional regulation in HOK cells. **(A)** Heat map showing differential peaks (log2 (normalized reads in peaks)) between NC, *IRF6*-MU, and *IRF6*-WT. **(B)** Volcano plots show the DEGs between *IRF6*-MU and *IRF6*-NC. The *x*-axis denotes log_2_FoldChange, and the *y*-axis indicates the-log_10_P_adj._ Genes significantly upregulated in *IRF6*-MU are marked in red, and genes significantly downregulated in epitope blue compared to control group. Moderated t-test (two-sided) with Benjamini–Hochberg correction. **(C)** GO analysis of predicted target genes of differentially expressed RNA. The top 10 biological process terms, cellular components, and molecular functions are shown.

To validate the influence of the newly discovered mutant loci on the functionality of the *IRF6* gene, the HOK cells infected with the lentivirus loaded with wild-type *IRF6* and mutant *IRF6* underwent RNA sequencing was conducted on HOK cells. Our findings revealed that there was a reduced number of DEGs in the HOK cells overexpressing the mutant IRF6 compared to the HOK cells that overexpressed wild-type IRF6 (Figure 3A). A profound transcriptomic shift was observed in the HOK cells harboring the mutant *IRF6*. Specifically, our analysis revealed 27 genes with increased expression and 38 with diminished expression in cells overexpressing the mutant *IRF6* gene (Figure 3B). This signified potential alterations in cellular signaling dynamics that were attributed to mutations in *IRF6*. Through comprehensive Gene Ontology (GO) analysis, we delineated that the mutated IRF6 primarily impacted the gene expressions integral to cell adhesion and epithelial cell migration, among other cellular functions (Figure 3C).

**FIGURE 4:**
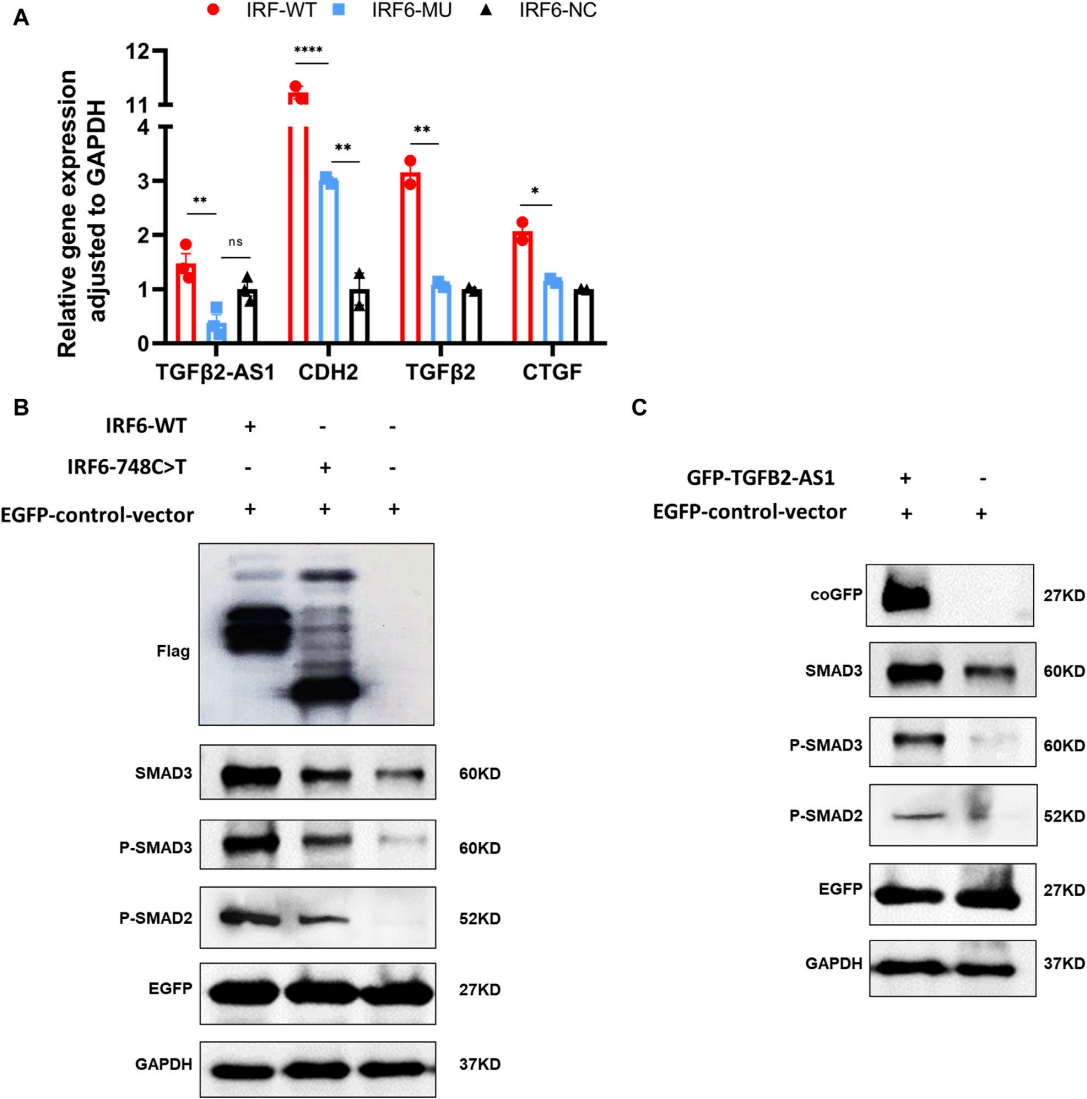
IRF6 and TGFβ2-AS1 regulate the TGFβ signaling pathway. **(A)** Bar graph presenting the relative expression levels of TGFβ2-AS1, CDH2, TGFβ2, and CTGF in HOK cells. The cells were categorized into three groups: HOK-*IRF6*-WT (wild type), HOK-*IRF6*-MU (mutant), and HOK-*IRF6*-NC (negative control for *IRF6* overexpression). **(B)** Western blot analysis showing the differences in protein levels of SMAD3, phosphorylated SMAD3 (P-SMAD3), and phosphorylated SMAD2 (P-SMAD2) between the cells exposed to the different treatments: *IRF6*-WT, *IRF6*-748C>T, control (negative control for *IRF6* overexpression). **(C)** Western blot analysis showing the differences in protein levels of SMAD3, phosphorylated SMAD3 (P-SMAD3), and phosphorylated SMAD2 (P-SMAD2) between the cells exposed to the different treatments: TGF*β*2-AS1 overexpressed, and control (negative control for TGF*β*2-AS1 overexpression). GAPDH was used as a loading control, and EGFP was used as a control for transfection efficiency. In all graphs, statistical significance is indicated by asterisks, with “**” denoting *p* < 0.01, “***” denoting *p* < 0.001, and “ns” indicating not significant.

By comparing the RNA-seq data between the mutant and wild-type groups, we observed a significant decrease in the expression of *TGFβ2-AS1*. Given that this lncRNA is involved in the TGFβ signaling pathway, we used qPCR to validate whether reduced expression of *TGFβ2-AS1* in HOK cells stably expressing either wild-type or mutant IRF6. Our findings revealed a notable reduction in the expression levels of *TGFβ2-AS1* in the cells expressing the mutant. Furthermore, there was a significant reduction in the expression levels of its downstream effectors, *CTGF* and *TGFβ2*, compared to the HOK cells overexpressing IRF6, yet there were no marked changes relative to the control group (Figure 4A).

To investigate the impact of IRF6-WT and IRF6-MU on the regulation of signaling pathways, we conducted Western blot analysis to assess their effects on the phosphorylation levels of SMAD2/3 (Figure 4B). In this experiment, HOK cells were transfected with an empty pCDH-SBP-HIS_8_ vector plasmid (control), and pCDH-*IRF6*-WT-flag-puro and pCDH-*IRF6*-MU-flag-puro plasmids (experimental groups) for comparison. Additionally, we co-transfected HOK cells with the pEGFP plasmid as an indicator to validate transfection efficiency. The results of Western blot revealed that IRF6 plays a role in enhancing the expression and phosphorylation of SMAD3 while also promoting the phosphorylation of SMAD2, effectively activating the TGFβ signaling pathway. Conversely, the p. R250X mutation in IRF6 was found to inhibit the expression of SMAD3 as well as the phosphorylation of both SMAD3 and SMAD2, thereby suppressing the activity of the signaling pathway. Similarly, HOK cells transfected with the pGreen-coGFP-puro vector plasmid served as a control group for comparison with cells transfected with the pGreen-coGFP-*TGFβ2-AS1*-puro plasmid. We observed that both the total and phosphorylated levels of SMAD3 and SMAD2 were higher in the cells overexpressing *TGFβ2-AS1* compared to the control group and were consistent with that of the corresponding levels in the cells overexpressing IRF6-p.R250X. This indicated that *TGFβ2-AS1* is a key component of the TGFβ pathway by promoting the expression and phosphorylation of SMAD3 and SMAD2 within the pathway.

**FIGURE 5:**
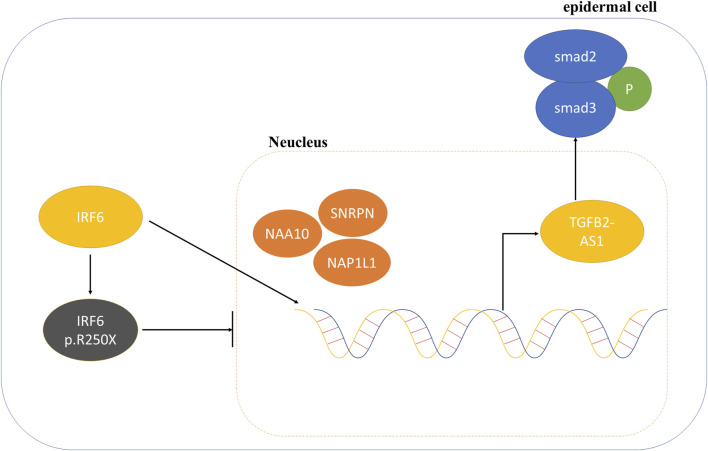
Schematic diagram of the mechanism involving *IRF6* and TGFβ2-AS1 in the TGFβ signaling pathway.

Consequently, we proposed that the newly identified mutation in IRF6 within the VWS lineage impaired its capacity to interact with proteins associated with histone modification. This impairment resulted in decreased TGFβ2-AS1 levels and inhibited the phosphorylation of SMAD3, which in turn critically regulated the activity of the TGFβ signaling pathway during embryogenesis. These molecular disruptions culminated in the pathogenesis observed in VWS (Figure 5).

## 4 Discussion

VWS is etiologically complex with both genetic and environmental contributions. The defects in patients with VWS occur during early embryological development (Murray, 2002). The *IRF6* gene that is implicated in the development of VWS is located in 1q32.2 and contains 10 exons, with the CDS region 1,404 bp in length encoding 467 amino acids. To date, 242 exonic mutations in *IRF6* have been revealed to be non-random, primarily occurring in exons 3, 4, 7, and 9 and accounting for 80% of all mutations (de Lima et al., 2009). This gene encodes a member of the *IRF* family, whose members share a highly-conserved N-terminal helix-turn-helix DNA-binding domain and a less-conserved C-terminal protein-binding domain. The encoded protein, IRF6, is a transcriptional activator (Parada-Sanchez et al., 2017; Alade et al., 2020; Tharuka et al., 2020). Mutations in this gene have also been known to cause VSS and popliteal pterygium syndrome (PSS) as well as non-syndromic orofacial cleft type (Kondo et al., 2002; Nouri et al., 2015; Neves et al., 2019).

To explore the etiological mutations of VWS, WES was performed on a family with inherited VWS. After WES and Sanger sequencing of peripheral blood samples from family members, a novel mutation (c.748C>T:p.R250X) in the *IRF6* gene (NCBI Refseq: NM_006147.4) was identified as the candidate pathogenic mutation. This mutation is a nonsense mutation resulting from a substitution of Arg250 residue, which is a conserved residue located in a functional domain. *IRF6*-c.748C>T:p.R250X affected the structure and function of IRF6 based on our modeling and prediction studies. Interestingly, this mutation was not observed in studies reporting on *IRF6* mutations in VWS cases, indicating that this mutation is novel. Different mutations in the DNA-binding domain and protein-binding domain are known to disrupt the function of IRF6. Exons 3 and 4 encode the DNA-binding domain, while exons 7 and 8 encode the protein-binding domain of IRF6 (Birkeland et al., 2011). The novel mutation (c.748C>T, p. R250X) was located within the SMIR protein-binding domain and led to the formation of a premature stop codon and a complete loss of the protein-binding domain (Kondo et al., 2002).

Nonsense-Mediated mRNA Decay (NMD) is a cellular regulatory mechanism that prevents the translation of aberrant transcripts by degrading mRNA transcripts containing premature termination codons (PTCs) (Lindeboom et al., 2016). Studies have shown that most mutant IRF6 transcripts are degraded by the NMD pathway, leading to haploinsufficiency of IRF6. Previous research identified that mutant IRF6 transcripts containing a PTC, which triggers NMD, are targeted for degradation. Specifically, this PTC is located in exon 7 of IRF6, more than 50–55 nucleotides away from the final exon-exon junction (Degen et al., 2020). The mutations found in this study were close to this region, suggesting that NMD serves as a regulatory mechanism for the expression of IRF6 by degrading mutant IRF6 mRNA transcripts, thereby causing IRF6 haploinsufficiency. This has significant implications for the pathogenesis of VWS.

To further explore the influence of the c.748C>T, p. R250X mutation on IRF6 function, an immunofluorescence assay was performed to compare the localization of the mutant IRF6 to wild-type IRF6 in cells. The results showed that wild-type IRF6 was mainly localized to the cytoplasm and the nucleus, while the mutated IRF6 (c.748C>T:p.R250X) largely accumulated in the cytoplasm. Based on the results of immunofluorescence, it was hypothesized that the mutations in *IRF6* possibly led to the inability of the gene to enter the nucleus, which in turn would prevent the activation of downstream signaling pathways and ultimately affect the normal function of the cell.

To evaluate the impact of a specific gene mutation, and therefore mutant protein, on cellular functions, we employed protein mass spectrometry to HOK cells stably expressing either wild-type or mutant *IRF6*. Our findings revealed variations in the binding capacity of the different proteins. Specifically, NAA10, SNRPN, and NAP1L1 had lower binding capacities to mutant IRF6 relative to wild-type IRF6, while the binding capacities of mutant IRF6 to MTHFD1, KDM1A, H2AFZ, and HDAC1 were higher compared to wild-type IRF6. Among the proteins with enhanced binding capacity, NAA10 encodes the catalytic subunit of NatA (Wu and Lyon, 2018). The literature has documented NAA10’s pivotal role in histone acetylation modifications, highlighting that its reduction or absence has detrimental consequences on embryonic development (Lee CC. et al., 2017). Furthermore, a notable connection exists between NAA10 and Runx2: overexpression of NAA10 results in the acetylation of Runx2, which subsequently influences BMP2 expression and, by extension, fetal maxillofacial development (Yoon et al., 2014).

Conversely, SNRPN and NAP1L1, which exhibited reduced binding capacities, have also been associated with affecting histone modifications (Lee JY. et al., 2017; Kim et al., 2017). A review of existing literature uncovered a crucial interaction between NAPILI and IRF6 in skin development. In particular, disruptions in the interactions between these proteins has been implicated in the potential causation of ankyloblepharon-ectodermal defects-cleft lip/palate (AEC) syndrome, suggesting that these proteins play a critical role in craniofacial development and highlighting the importance of their normal function for preventing developmental disorders (Mollo et al., 2015). NAA10 has been shown to function abnormally in maxillofacial development, leading to conditions such as microcephaly and abnormal epidermal development (Wu and Lyon, 2018). Furthermore, NAA10 participates in the TGFβ signaling pathway and is involved in embryonic development during EMT during embryonic development (Lv et al., 2021; Le et al., 2023).

Similarly, SNRPN’s role in facial development has been documented, with its dysfunction associated with craniofacial anomalies like mandibulofacial dysostosis Guion-Almeida type and cerebrocostomandibular syndrome (Wood et al., 2019; Alam et al., 2022).

MTHFD1 anomalies were shown to induce alterations in maternal total folate levels, disrupt one-carbon metabolism, and elevate the prevalence of developmental defects (Christensen et al., 2021). KDM1A, H2AFZ, and HDAC1 have been shown to be integral to ensuring proper histone modification. KDM1A has been associated with histone demethylation and functions either as a transcriptional co-activator or co-repressor in histone 3 lysine 4 and 9 (H3K4/9) demethylation. Its involvement is also noted in the Wnt/β-catenin signaling pathway, a critical pathway associated with maxillofacial development (Song et al., 2022; Zhang et al., 2022). H2AFZ plays a crucial role in encoding H2A.Z.1, which is indispensable in numerous cellular biological processes, including gene expression and genomic stability. A deletion of H2AFZ culminates in G1 phase arrest and cellular senescence (Sales-Gil et al., 2021).

Our investigation revealed that *IRF6* mutations markedly influence *TGFβ2-AS1* expression. Prior research has established that *IRF6*, whose expression is modulated by upstream *TGFβ3*, affects the TGFβ pathway through downstream *TGFβ2*, thereby playing a crucial role in palatal fusion during embryogenesis (Ke et al., 2015). We observed using qPCR that *IRF6* mutations led to a reduction in the mRNA expression levels of *TGFβ2-AS1*, *TGFβ2*, and *CTGF* compared to in the cells overexpressing wild-type IRF6, although these alterations did not significantly diverge from the control samples. Within the TGFβ signaling cascade, *IRF6* mutations altered the expression and phosphorylation of downstream SMAD2/3, thereby modulating the functionality of the TGFβ pathway. Moreover, *TGFβ2-AS1* has been shown to promote the phosphorylation of SMAD2/3. Previous research highlights the bidirectional regulatory capacity of this lncRNA within the TGFβ signaling framework, showcasing its ability to both attenuate and intensify gene responses elicited by TGFβ activation. This dual functionality underscores *TGFβ2-AS1*’s pivotal role as a modulator within this pathway (Conway and Kaartinen, 2011; Nakajima et al., 2018). Additionally, *TGFβ2-AS1* plays a role in cellular morphology, particularly influencing stress fiber formation and cell adhesion dynamics. Such contributions are indicative of its significant role in cytoskeletal organization and cellular interactions, which are imperative for craniofacial development and structural integrity (David and Massague, 2018). Consequently, our research has unearthed a novel function for *TGFβ2-AS1* in the etiology of VWS, acting as a key regulatory element within the TGFβ signaling pathway. Governed by IRF6, it modulates the TGFβ pathway’s activity by impacting SMAD2 and SMAD3 phosphorylation, ultimately affecting craniofacial morphogenesis. SMAD2 and SMAD3 are normally translocated to the nucleus after being phosphorylated to exert transcriptional regulation (Kushioka et al., 2020). However, this phosphorylated state may also increase their interaction with SMURF2. SMURF (SMAD-specific ubiquitin ligase) family proteins are E3 ubiquitin ligases, which function *in vivo* primarily by regulating ubiquitin-dependent protein degradation. Several studies have shown that upon binding of phosphorylated SMAD2/3 to SMURF2, SMURF2 can promote their ubiquitination, which can lead to their degradation and reduce the duration and strength of signalling (Keyan et al., 2023). However, SMURF2 preferentially targets Smad1 for ubiquitination and degradation, having a much weaker effect on Smad2 protein levels, but not Smad3 levels (Zhang et al., 2001).

The epithelial-to-mesenchymal transition (EMT) plays a crucial role in craniofacial development, particularly through the migration and differentiation of cranial neural crest cells (CNCCs). These cells are essential in shaping the vertebrate head during embryogenesis, originating from multipotent neural crest cells (Mandalos et al., 2023). Identified as transient embryonic stem cells, neural crest cells (NCCs) are fundamental to the development of craniofacial bone, cartilage, sensory systems, and cranial nerves. Their rapid EMT and subsequent migration are governed by dynamic changes in gene expression and protein localization, illustrating a highly regulated process crucial for embryonic development (Kim et al., 2024). IRF6’s involvement in craniofacial EMT highlights a complex regulatory network. Through pathways involving TGFβ3 and TWIST1, IRF6 influences the behavior of cranial neural crest cells, affecting the development of key tissues like the palate. This underscores IRF6’s significant role in cellular differentiation, migration, and overall craniofacial embryonic development, orchestrating the sophisticated processes of facial formation and organogenesis (Ke et al., 2015; Bertol et al., 2022). Based on the results from immunofluorescence localization, we deduced that the novel mutant IRF6 had a reduced ability to enter the cell nucleus. This reduction in nuclear localization led to a decreased binding to histones such as NAA10, SNRPN, and NAPILI, impairing the EMT process and also disrupting the TGFβ signaling pathway. Consequently, this impairment affects craniofacial development, resulting in developmental defects. This interpretation highlights the critical role of proper IRF6 function in cellular localization and interaction with histones for the maintenance of normal EMT processes, which are essential for craniofacial development.

## 5 Conclusion

In summary, a novel nonsense mutation—c.748C>T:p.R250X—in the *IRF6* gene was identified in a three-generation family with VWS, which has not previously been reported. This discovery underscores the association between IRF6 and the VWS phenotype, supporting the pathogenetic role of the *IFR6* mutation in the development of VWS and the key role played by this gene in orofacial development. The c.748C>T:p.R250X mutation disrupted the normal function of IRF6 and affected the cellular localization of IRF6 and the interactions that it has with other proteins, such as histones. This study has also shed light on the role of *TGFβ2-AS1*, an lncRNA that modulates the TGFβ signaling pathway by influencing SMAD2/3 phosphorylation, a key player in maxillofacial development. The identification of this RNA’s bidirectional influence on gene expression within the TGFβ pathway enhances our understanding of the molecular mechanisms underpinning VWS, paving the way for advancements in prevention, treatment, and prognosis.

## Data Availability

The data that support the findings of this study are available from the corresponding author upon reasonable request. RNA-seq and mass spectrometry results can be found in the Supplementary Material.

## References

[B1] Alade A. A. Buxo-Martinez C. J. Mossey P. A. Gowans L. J. J. Eshete M. A. Adeyemo W. L. (2020). Non-random distribution of deleterious mutations in the DNA and protein-binding domains of IRF6 are associated with Van Der Woude syndrome. Mol. Genet. Genomic Med. 8 (8), e1355. 10.1002/mgg3.1355 32558391 PMC7434609

[B2] Alam S. S. Kumar S. Beauchamp M. C. Bareke E. Boucher A. Nzirorera N. (2022). Snrpb is required in murine neural crest cells for proper splicing and craniofacial morphogenesis. Dis. Model Mech. 15 (6), dmm049544. 10.1242/dmm.049544 35593225 PMC9235875

[B3] AlMegbel A. M. Shuler C. F. (2020). SMAD2 overexpression rescues the TGF-β3 null mutant mice cleft palate by increased apoptosis. Differentiation 111, 60–69. 10.1016/j.diff.2019.10.001 31677482

[B4] Beaty T. H. Murray J. C. Marazita M. L. Munger R. G. Ruczinski I. Hetmanski J. B. (2010). A genome-wide association study of cleft lip with and without cleft palate identifies risk variants near MAFB and ABCA4. Nat. Genet. 42 (6), 525–529. 10.1038/ng.580 20436469 PMC2941216

[B5] Bennun R. D. Stefano E. Moggi L. E. (2018). Van der Woude and Popliteal Pterygium Syndromes. J. Craniofac Surg. 29 (6), 1434–1436. 10.1097/SCS.0000000000004698 29916977

[B6] Bertol J. W. Johnston S. Ahmed R. Xie V. K. Hubka K. M. Cruz L. (2022). TWIST1 interacts with β/δ-catenins during neural tube development and regulates fate transition in cranial neural crest cells. Development 149 (15), dev200068. 10.1242/dev.200068 35781329 PMC9440756

[B7] Birkeland A. C. Larrabee Y. Kent D. T. Flores C. Su G. H. Lee J. H. (2011). Novel IRF6 mutations in Honduran Van der Woude syndrome patients. Mol. Med. Rep. 4 (2), 237–241. 10.3892/mmr.2011.423 21468557

[B8] Busche A. Hehr U. Sieg P. Gillessen-Kaesbach G. (2016). Van der Woude and Popliteal Pterygium Syndromes: Broad intrafamilial variability in a three generation family with mutation in IRF6. Am. J. Med. Genet. A 170 (9), 2404–2407. 10.1002/ajmg.a.37791 27286731

[B9] Charzewska A. Obersztyn E. Hoffman-Zacharska D. Lenart J. Poznanski J. Bal J. (2015). Novel mutations in the IRF6 gene on the background of known polymorphisms in polish patients with orofacial clefting. Cleft Palate Craniofac J. 52 (5), e161–e167. 10.1597/14-030 25489771

[B10] Christensen K. E. Malysheva O. V. Carlin S. Matias F. MacFarlane A. J. Jacobs R. L. (2021). Mild choline deficiency and MTHFD1 synthetase deficiency interact to increase incidence of developmental delays and defects in mice. Nutrients 14 (1), 127. 10.3390/nu14010127 35011003 PMC8747146

[B11] Conway S. J. Kaartinen V. (2011). TGFβ superfamily signaling in the neural crest lineage. Cell Adh Migr. 5 (3), 232–236. 10.4161/cam.5.3.15498 21436616 PMC3210207

[B12] Cui R. Chen D. Li N. Cai M. Wan T. Zhang X. (2022). PARD3 gene variation as candidate cause of nonsyndromic cleft palate only. J. Cell Mol. Med. 26 (15), 4292–4304. 10.1111/jcmm.17452 35789100 PMC9344820

[B13] David C. J. Massague J. (2018). Contextual determinants of TGFβ action in development, immunity and cancer. Nat. Rev. Mol. Cell Biol. 19 (7), 419–435. 10.1038/s41580-018-0007-0 29643418 PMC7457231

[B14] Degen M. Girousi E. Feldmann J. Parisi L. La Scala G. C. Schnyder I. (2020). A Novel Van der Woude Syndrome-Causing IRF6 Variant Is Subject to Incomplete Non-sense-Mediated mRNA Decay Affecting the Phenotype of Keratinocytes. Front. Cell Dev. Biol. 8, 583115. 10.3389/fcell.2020.583115 33117810 PMC7552806

[B15] de Lima R. L. Hoper S. A. Ghassibe M. Cooper M. E. Rorick N. K. Kondo S. (2009). Prevalence and nonrandom distribution of exonic mutations in interferon regulatory factor 6 in 307 families with Van der Woude syndrome and 37 families with popliteal pterygium syndrome. Genet. Med. 11 (4), 241–247. 10.1097/GIM.0b013e318197a49a 19282774 PMC2789395

[B16] Ke C. Y. Mei H. H. Wong F. H. Lo L. J. (2019). IRF6 and TAK1 coordinately promote the activation of HIPK2 to stimulate apoptosis during palate fusion. Sci. Signal 12 (593), eaav7666. 10.1126/scisignal.aav7666 31387937

[B17] Ke C. Y. Xiao W. L. Chen C. M. Lo L. J. Wong F. H. (2015). IRF6 is the mediator of TGFβ3 during regulation of the epithelial mesenchymal transition and palatal fusion. Sci. Rep. 5, 12791. 10.1038/srep12791 26240017 PMC4523936

[B18] Keyan K. S. Salim S. Gowda S. Abdelrahman D. Amir S. S. Islam Z. (2023). Control of TGFβ signalling by ubiquitination independent function of E3 ubiquitin ligase TRIP12. Cell Death Dis. 14 (10), 692. 10.1038/s41419-023-06215-y 37863914 PMC10589240

[B19] Kim I. K. Diamond M. S. Yuan S. Kemp S. B. Kahn B. M. Li Q. (2024). Plasticity-induced repression of Irf6 underlies acquired resistance to cancer immunotherapy in pancreatic ductal adenocarcinoma. Nat. Commun. 15 (1), 1532. 10.1038/s41467-024-46048-7 38378697 PMC10879147

[B20] Kim Y. Lee H. M. Xiong Y. Sciaky N. Hulbert S. W. Cao X. (2017). Targeting the histone methyltransferase G9a activates imprinted genes and improves survival of a mouse model of Prader-Willi syndrome. Nat. Med. 23 (2), 213–222. 10.1038/nm.4257 28024084 PMC5589073

[B21] Kondo S. Schutte B. C. Richardson R. J. Bjork B. C. Knight A. S. Watanabe Y. (2002). Mutations in IRF6 cause Van der Woude and popliteal pterygium syndromes. Nat. Genet. 32 (2), 285–289. 10.1038/ng985 12219090 PMC3169431

[B22] Kushioka J. Kaito T. Okada R. Ishiguro H. Bal Z. Kodama J. (2020). A novel negative regulatory mechanism of Smurf2 in BMP/Smad signaling in bone. Bone Res. 8 (1), 41. 10.1038/s41413-020-00115-z 33298874 PMC7680794

[B23] Le M. K. Vuong H. G. Nguyen T. T. T. Kondo T. (2023). NAA10 overexpression dictates distinct epigenetic, genetic, and clinicopathological characteristics in adult gliomas. J. Neuropathol. Exp. Neurol. 82 (7), 650–658. 10.1093/jnen/nlad037 37253389

[B24] Lee C. C. Peng S. H. Shen L. (2017a). The role of N-alpha-acetyltransferase 10 protein in DNA methylation and genomic imprinting. Mol. Cell 68 (1), 89–103 e107. 28943313 10.1016/j.molcel.2017.08.025 PMC6322414

[B25] Lee J. Y. Lake R. J. Kirk J. Bohr V. A. Fan H. Y. Hohng S. (2017b). NAP1L1 accelerates activation and decreases pausing to enhance nucleosome remodeling by CSB. Nucleic Acids Res. 45 (8), 4696–4707. 10.1093/nar/gkx188 28369616 PMC5416873

[B26] Leslie E. J. Standley J. Compton J. Bale S. Schutte B. C. Murray J. C. (2013). Comparative analysis of IRF6 variants in families with Van der Woude syndrome and popliteal pterygium syndrome using public whole-exome databases. Genet. Med. 15 (5), 338–344. 10.1038/gim.2012.141 23154523 PMC3723330

[B27] Lindeboom R. G. Supek F. Lehner B. (2016). The rules and impact of nonsense-mediated mRNA decay in human cancers. Nat. Genet. 48 (10), 1112–1118. 10.1038/ng.3664 27618451 PMC5045715

[B28] Lv S. Luo T. Yang Y. Li Y. Yang J. Xu J. (2021). Naa10p and IKKα interaction regulates EMT in oral squamous cell carcinoma via TGF-β1/Smad pathway. J. Cell Mol. Med. 25 (14), 6760–6772. 10.1111/jcmm.16680 34060226 PMC8278082

[B29] Malik S. Wilcox E. R. Naz S. (2014). Novel lip pit phenotypes and mutations of IRF6 in Van der Woude syndrome patients from Pakistan. Clin. Genet. 85 (5), 487–491. 10.1111/cge.12207 23713753

[B30] Mandalos N. P. Dimou A. Gavala M. A. Lambraki E. Remboutsika E. (2023). Craniofacial development is fine-tuned by Sox2. Genes (Basel) 14 (2), 380. 10.3390/genes14020380 36833308 PMC9956624

[B31] Mollo M. R. Antonini D. Mitchell K. Fortugno P. Costanzo A. Dixon J. (2015). p63-dependent and independent mechanisms of nectin-1 and nectin-4 regulation in the epidermis. Exp. Dermatol 24 (2), 114–119. 10.1111/exd.12593 25387952 PMC4329386

[B32] Murray J. C. (2002). Gene/environment causes of cleft lip and/or palate. Clin. Genet. 61 (4), 248–256. 10.1034/j.1399-0004.2002.610402.x 12030886

[B33] Nakajima A. Shuler C. F. Gulka A. O. D. Hanai J. I. (2018). TGF-Β signaling and the epithelial-mesenchymal transition during palatal fusion. Int. J. Mol. Sci. 19 (11), 3638. 10.3390/ijms19113638 30463190 PMC6274911

[B34] Neves L. T. Dionisio T. J. Garbieri T. F. Parisi V. A. Oliveira F. V. Oliveira T. M. (2019). Novel rare variations in IRF6 in subjects with non-syndromic cleft lip and palate and dental agenesis. Oral Dis. 25 (1), 223–233. 10.1111/odi.12975 30195270

[B35] Nouri N. Memarzadeh M. Carinci F. Cura F. Scapoli L. (2015). Family-based association analysis between nonsyndromic cleft lip with or without cleft palate and IRF6 polymorphism in an Iranian population. Clin. Oral Investig. 19 (4), 891–894. 10.1007/s00784-014-1305-3 25220223

[B36] Papoutsoglou P. Tsubakihara Y. Caja L. (2019). The TGFB2-AS1 lncRNA regulates TGF-beta signaling by modulating corepressor activity. Cell Rep. 28 (12), 3182–3198 e3111. 10.1016/j.celrep.2019.08.028 31533040 PMC6859500

[B37] Parada-Sanchez M. T. Chu E. Y. Cox L. L. Undurty S. S. Standley J. M. Murray J. C. (2017). Disrupted IRF6-NME1/2 complexes as a cause of cleft lip/palate. J. Dent. Res. 96 (11), 1330–1338. 10.1177/0022034517723615 28767310 PMC5613882

[B38] Peyrard-Janvid M. Leslie E. J. Kousa Y. A. Smith T. L. Dunnwald M. Magnusson M. (2014). Dominant mutations in GRHL3 cause Van der Woude Syndrome and disrupt oral periderm development. Am. J. Hum. Genet. 94 (1), 23–32. 10.1016/j.ajhg.2013.11.009 24360809 PMC3882735

[B39] Sales-Gil R. Kommer D. C. de Castro I. J. Amin H. A. Vinciotti V. Sisu C. (2021). Non-redundant functions of H2A.Z.1 and H2A.Z.2 in chromosome segregation and cell cycle progression. EMBO Rep. 22 (11), e52061. 10.15252/embr.202052061 34423893 PMC8567233

[B40] Song Y. Zhang H. Yang X. Shi Y. Yu B. (2022). Annual review of lysine-specific demethylase 1 (LSD1/KDM1A) inhibitors in 2021. Eur. J. Med. Chem. 228, 114042. 10.1016/j.ejmech.2021.114042 34915312

[B41] Starink E. Hokken-Koelega A. C. S. Visser T. J. Baan J. Peeters R. P. de Graaff L. C. G. (2017). Genetic analysis of IRF6, a gene involved in craniofacial midline formation, in relation to pituitary and facial morphology of patients with idiopathic growth hormone deficiency. Pituitary 20 (5), 499–508. 10.1007/s11102-017-0808-8 28593555 PMC5606942

[B42] Tharuka M. D. N. Yang H. Lee J. (2020). Expression, subcellular localization, and potential antiviral function of three interferon regulatory factors in the big-belly seahorse (*Hippocampus abdominalis*). Fish. Shellfish Immunol. 96, 297–310. 10.1016/j.fsi.2019.11.026 31811886

[B43] Van Der Woude A. (1954). Fistula labii inferioris congenita and its association with cleft lip and palate. Am. J. Hum. Genet. 6 (2), 244–256. 13158329 PMC1716548

[B44] Wang T. J. Hsieh K. S. Lai J. P. Tsai M. H. Liang Y. C. Chang Y. H. (2019). Novel mutations of IRF6 gene in Taiwanese Van der Woude syndrome patients. Pediatr. Neonatol. 60 (2), 218–220. 10.1016/j.pedneo.2018.04.008 30982524

[B45] Wood K. A. Rowlands C. F. Qureshi W. M. S. Thomas H. B. Buczek W. A. Briggs T. A. (2019). Disease modeling of core pre-mRNA splicing factor haploinsufficiency. Hum. Mol. Genet. 28 (22), 3704–3723. 10.1093/hmg/ddz169 31304552 PMC6935387

[B46] Wu Y. Lyon G. J. (2018). NAA10-related syndrome. Exp. Mol. Med. 50 (7), 1–10. 10.1038/s12276-018-0098-x PMC6063861 30054457

[B47] Yoon H. Kim H. L. Chun Y. S. Shin D. H. Lee K. H. Shin C. S. (2014). NAA10 controls osteoblast differentiation and bone formation as a feedback regulator of Runx2. Nat. Commun. 5, 5176. 10.1038/ncomms6176 25376646

[B48] Yu Y. Wan Y. Qin C. Yue H. Bian Z. He M. (2020). Novel IRF6 mutations in Chinese Han families with Van der Woude syndrome. Mol. Genet. Genomic Med. 8 (5), e1196. 10.1002/mgg3.1196 32108996 PMC7216816

[B49] Zhang W. Ruan X. Li Y. Zhi J. Hu L. Hou X. (2022). KDM1A promotes thyroid cancer progression and maintains stemness through the Wnt/β-catenin signaling pathway. Theranostics 12 (4), 1500–1517. 10.7150/thno.66142 35198054 PMC8825597

[B50] Zhang Y. Chang C. Gehling D. J. Hemmati-Brivanlou A. Derynck R. (2001). Regulation of Smad degradation and activity by Smurf2, an E3 ubiquitin ligase. Proc. Natl. Acad. Sci. U. S. A. 98 (3), 974–979. 10.1073/pnas.98.3.974 11158580 PMC14694

[B51] Zhao H. Zhang M. Zhong W. Zhang J. Huang W. Zhang Y. (2018). A novel IRF6 mutation causing non-syndromic cleft lip with or without cleft palate in a pedigree. Mutagenesis 33 (3), 195–202. 10.1093/mutage/gey012 30053123

